# A New Curing-Dependent Viscoelastic Constitutive Model and Stress-Increment Equations for Numerical Simulation Predicting the Cure-Induced Stress of Epoxy Resin

**DOI:** 10.3390/polym18151844

**Published:** 2026-07-28

**Authors:** Qi Luo, Guo Li, Yibo Wu, Hongbo Tao, Shuailong Zhou, Mi Xu, Anxin Ding

**Affiliations:** 1 Luoyang Ship Material Research Institute, Luoyang 471023, China18237937656@139.com (H.T.);; 2 School of Materials Science and Engineering, Wuhan University of Technology, Wuhan 430070, China

**Keywords:** viscoelastic constitutive model, cure-induced stress, thermo-rheologically complex materials, finite element simulation

## Abstract

A cure-dependent viscoelastic constitutive model is developed to describe the stress response of epoxy resin during curing. The progressive formation of the load-carrying network is represented phenomenologically through cure-dependent equilibrium and Maxwell-branch stiffness functions. A one-dimensional history-integral equation is derived and extended to non-isothermal conditions by incorporating temperature-dependent stiffness and reduced-time effects. Explicit stress-increment equations are then obtained for one-dimensional and three-dimensional isotropic materials and implemented in ABAQUS through a UMAT. Two idealized numerical cases are used to verify the consistency between the UMAT results and direct constitutive calculations. An illustrative encapsulation model further shows that different assumptions regarding the curing-dependent stiffness factor produce substantial differences in peak and final stresses. An additional cooling-rate sensitivity analysis shows that increasing the cooling rate increases the magnitude of the final compressive stress because less time is available for viscoelastic relaxation. A formal tensorial extension is also provided for anisotropic thermosetting materials, although it is not numerically assessed. The present results constitute constitutive and numerical verification rather than material-specific experimental validation.

## 1. Introduction

Epoxy resins are widely used to encapsulate electronic devices, integrated circuits, and light-emitting diodes (LEDs) [1,2,3] due to high heat resistance, superior adhesion, excellent electrical insulation, and impressive corrosion resistance. The encapsulation process typically involves injecting uncured liquid resin into the cavity of the component, which then solidifies through a curing reaction. This curing process is fundamental to achieving the desired material properties, as it involves the formation of new chemical bonds both within and between molecules, leading to a solidified, durable material. The curing reaction of epoxy resin is not only exothermic, generating heat during the reaction [4,5,6], but also accompanied by curing shrinkage [7,8,9,10,11]. The heat released elevates the resin temperature, inducing thermal strain, while curing shrinkage contributes to overall deformation. These factors make thermal deformation and curing shrinkage primary contributors to the cure-induced deformation of epoxy resin. Recent studies reveal that the interplay of thermal expansion and chemical shrinkage over the entire curing cycle strongly depends on the degree of cure and the presence of additives [12]. In scenarios where the curing process occurs at uniform temperature conditions without external constraints, the resin may undergo free-curing deformation, which typically does not induce residual stresses. However, when complex geometries or component configurations constrain the curing process, internal stresses can develop within the encapsulated electronic components [10]. Excessive internal stress can compromise the performance of encapsulated components and lead to cracking of the epoxy resin, thus underscoring the importance of designing encapsulation processes that minimize cure-induced residual stresses. Cure-induced deformation is a significant source of molding-induced stress in thermosetting composite materials [13,14,15,16], highlighting the importance of investigating the stress of epoxy resin during curing. Current methodologies employ finite element simulation technology to predict the distribution and evolution of stress during curing under various constrained conditions. These simulations are invaluable for optimizing production processes and enhancing product quality in a cost-effective manner [17,18,19,20,21,22,23,24].

To predict cure-induced stress accurately, it is necessary to establish a curing-kinetics model to determine the degree of cure, a heat-conduction model to calculate the temperature field, and a mechanical constitutive model to evaluate the resulting stress. Extensive studies have been conducted on cure kinetics and heat transfer during epoxy curing [3,4,5,6,11,25,26,27,28]. Mechanical constitutive models are equally important because they define the relationship between strain history and stress development during the liquid-to-solid transition [29,30]. Purely viscous or elastic descriptions cannot adequately represent this process, whereas viscoelastic models provide a unified framework for describing both fluid-like and solid-like responses [31].

The generalized Maxwell model is widely used to characterize the relaxation behavior of fully cured polymers [32,33,34]. During curing, however, both the fully relaxed and unrelaxed moduli evolve continuously with the degree of cure [35,36,37], while the shift factor is affected by both temperature and cure state [38]. Ding et al. [39] developed a viscoelastic constitutive model and stress-increment equation for thermo-rheologically complex materials by incorporating temperature-dependent effects. Nevertheless, the irreversible evolution of the load-carrying network during curing was not explicitly considered. Yang et al. [35] established a relationship between shear relaxation behavior and the degree of cure, but did not derive a complete history-dependent stress–strain relationship or explicit stress-increment equations for finite element implementation. Hossain et al. [37] interpreted the evolution of modulus during curing through changes in network stiffness, providing an important physical basis for describing cure-dependent viscoelasticity. More recently, in situ sensing combined with numerical simulation has been used to characterize curing-induced interfacial stresses [40], statistically based viscoelastic models have been developed to describe the evolution of glass-forming polymer networks [41], and process-integrated multiscale models have been proposed for warpage prediction in epoxy molding compounds [42]. Despite these advances, an explicit constitutive framework is still required to describe how stiffness contributions formed at different cure stages enter the stress history and to provide corresponding stress-increment equations suitable for finite element implementation.

In this paper, a curing-dependent viscoelastic constitutive model for thermo-rheologically complex materials is developed to calculate the cure-induced stress of epoxy resin. The progressive evolution of the load-carrying network is represented phenomenologically through cure-dependent equilibrium and Maxwell-branch stiffness functions. Based on this representation, a one-dimensional integral-type constitutive equation is derived and further extended to non-isothermal curing by incorporating temperature-dependent stiffness parameters and reduced-time effects. Explicit stress-increment equations are then obtained for one-dimensional and three-dimensional isotropic materials and implemented in finite element calculations. Their numerical consistency is verified by comparison with direct constitutive calculations. The influences of both the curing-dependent stiffness-factor assumption and the prescribed cooling rate on the calculated encapsulation stress are subsequently examined. A formal tensorial extension is also presented for anisotropic thermosetting materials, although its numerical assessment is beyond the scope of the present study.

## 2. Theoretical Model

### 2.1. The One-Dimensional Viscoelastic Model for the Isothermal Curing Process

**Figure 1:**
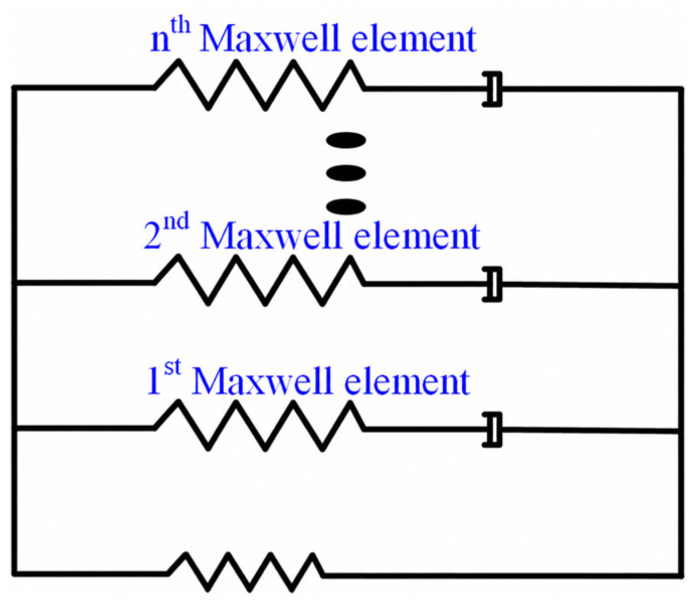
The generalized Maxwell model. The labels “1st”, “2nd”, and “n-th” identify the Maxwell branches; the ellipsis denotes the omitted intermediate branches; and the lower spring represents the equilibrium elastic branch.

The generalized Maxwell model represents the viscoelasticity of one-dimensional materials; its one-dimensional relaxation modulus can be expressed as:

(1) $$E(t)=E^{\infty }+\left(E^{0}-E^{\infty }\right)\sum_{k=1}^{n}W_{k}\exp(-\frac{t}{a_{T}\tau_{k}})$$

where $E^{\infty }$ and $E^{0}$ are the fully relaxed and unrelaxed moduli, respectively; $W_{k}$ is the weight factor; $a_{T}$ is the shift factor and $\tau_{k}$ represents the relaxation time. The mathematical representation of viscoelasticity can be expressed in differential and integral forms. The differential form of the viscoelastic constitutive relationship for the generalized Maxwell model in Figure 1 is given by Equation (2), while the integral form is represented by Equation (3) [43].

(2) $$\sum_{i=1}^{n}{\dot{\sigma }}_{i}=\sum_{i=1}^{n}\left(k_{i}\dot{\varepsilon }-\frac{\sigma_{i}k_{i}}{\eta_{i}}\right)$$

(3) $$\sigma (t)=\int_{0}^{t}E(t-\tau )\frac{d\varepsilon }{d\tau }d\tau$$

where the subscript *i* denotes the Maxwell element index, $\dot{\sigma }$ represents the first-order time derivative of the stress in the Maxwell element, $\dot{\varepsilon }$ represents the first-order time derivative of the mechanical strain, $k_{i}$ represents the spring modulus, and $\eta_{i}$ represents the dashpot viscosity. The specific expression of E(t) is Equation (1). Unless otherwise specified, all strain symbols mentioned in this paper refer to mechanical strain.

The prerequisite for the widespread use of these differential and integral viscoelastic constitutive equations is that the generalized Maxwell model in Figure 1 is only subjected to external forces. At the same time, its structure remains unchanged, such as when the model is stretched or compressed. However, the generalized Maxwell model with a fixed structure, as shown in Figure 1, is no longer applicable to epoxy resin undergoing curing because it cannot adequately describe the relationship between the stress increment and the mechanical strain increment during curing. The curing reaction of epoxy resin leads to a gradual change in viscoelasticity. How the generalized Maxwell model reflects this change in the viscoelasticity of epoxy resin will be discussed in the following sections.

**Figure 2:**
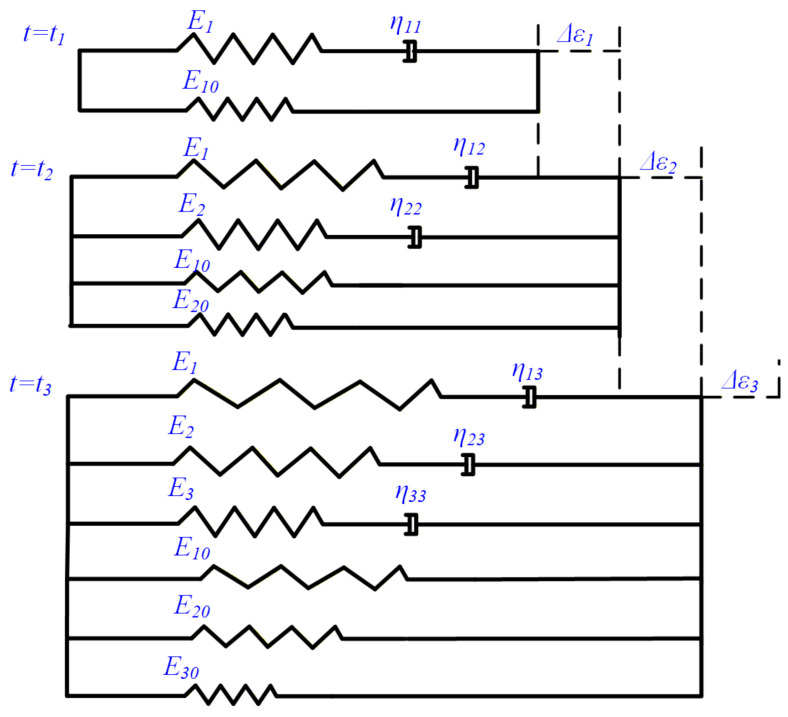
Phenomenological network-cohort representation of the one-dimensional curing process. Here, $E_{i}$ and $E_{i0}$ denote the stiffnesses of the Maxwell spring and equilibrium spring, respectively; $\eta_{ij}$ denotes the dashpot viscosity; $\Delta \varepsilon_{j}$ is the strain increment applied at $t_{j}$; and the dashed lines indicate the corresponding strain-increment positions.

The curing reaction of epoxy resin involves the formation of new chemical bonds within and between molecules, leading to irreversible changes in the macroscopic mechanical properties of the resin. To accurately capture this change process in the viscoelastic model, a theoretical framework is proposed that incorporates additional Maxwell elements and springs into the generalized Maxwell model to account for the formation of new chemical bonds. Figure 2 illustrates the changes in an individual Maxwell element and the stress evolution process in the generalized Maxwell model during isothermal curing. As the number of parallel Maxwell elements and springs increases, the macroscopic mechanical properties of the epoxy resin change. The strain increment progressively affects only those Maxwell elements and springs that were present before the increment.

The progressive introduction of additional Maxwell and equilibrium branches is a phenomenological representation of network formation rather than a literal description of molecular springs and dashpots. Each newly activated branch represents an incremental population, or cohort, of a polymer network that becomes mechanically active as crosslinking proceeds. The increasing number and stiffness of the active contributions therefore represent the macroscopic buildup of the load-carrying network.

Each network cohort is assumed to adopt the material configuration at its activation time as its own stress-free reference configuration. Consequently, it does not inherit mechanical strain accumulated before activation. Once activated, however, it responds to all subsequent strain increments. In the discrete construction shown in Figure 2, this assumption means that the branch activated at $t_{j}$ carries no stress at $t_{j}$, whereas strain increments applied after $t_{j}$ contribute to its stress. This evolving-reference-state assumption is the origin of the cure-history dependence in the proposed formulation. This assumption is intended primarily for the load-bearing post-gel regime, and its applicability should be assessed using material-specific experimental data.

At $t_{1}$, a Maxwell element in conjunction with a spring represents a one-dimensional viscoelastic model, and the model experiences a strain increment $\Delta \varepsilon_{1}$. From $t_{1}$ to $t_{2}$, its stress is:

(4) $$\delta (t)=E_{10}\Delta \varepsilon_{1}+E_{1}\Delta \varepsilon_{1}\exp(-\frac{t-t_{1}}{\tau_{1}})$$

where $\tau_{1}$ is the relaxation time, and $\tau_{1}=E_{1}/\eta_{1}$.

At $t_{2}$, as the degree of cure increases, a new Maxwell element and spring are added alongside the original ones. The newly activated branch is assumed to be stress-free at $t_{2}$ and therefore does not inherit the strain history preceding its activation. All strain increments applied after $t_{2}$ act on this branch. The stress from time $t_{2}$ to time $t_{3}$ is:

(5) $$\begin{array}{l} \delta (t)=E_{10}\Delta \varepsilon_{1}+E_{10}\Delta \varepsilon_{2}+E_{1}\Delta \varepsilon_{1}\exp(-\frac{\xi^{t}-\xi^{t_{1}}}{\tau_{1}})+E_{1}\Delta \varepsilon_{2}\exp(-\frac{\xi^{t}-\xi^{t_{2}}}{\tau_{1}})\\ \;\;+E_{20}\Delta \varepsilon_{2}+E_{2}\Delta \varepsilon_{2}\exp(-\frac{\xi^{t}-\xi^{t_{2}}}{\tau_{2}}) \end{array}$$

where $\xi^{t}$ is the reduction time, calculated using Equation (6); $a_{\left(T,\alpha \right)}$ is the shift factor influenced by temperature *T* and degree of cure *α*.

(6) $$\xi^{t}=\int_{0}^{t}\frac{1}{a_{\left(T,\alpha \right)}}dt$$

At $t_{3}$, there are three Maxwell elements and three springs, and the entire model has a strain increment $\Delta \varepsilon_{3}$. After $t_{3}$, the stress at time t is:

(7) $$\begin{array}{l} \delta (t)=E_{10}\Delta \varepsilon_{1}+E_{10}\Delta \varepsilon_{2}+E_{10}\Delta \varepsilon_{3}\\ \;\;+E_{1}\Delta \varepsilon_{1}\exp(-\frac{\xi^{t}-\xi^{t_{1}}}{\tau_{1}})+E_{1}\Delta \varepsilon_{2}\exp(-\frac{\xi^{t}-\xi^{t_{2}}}{\tau_{1}})+E_{1}\Delta \varepsilon_{3}\exp(-\frac{\xi^{t}-\xi^{t_{3}}}{\tau_{1}})\\ \;\;+E_{20}\Delta \varepsilon_{2}+E_{20}\Delta \varepsilon_{3}+E_{2}\Delta \varepsilon_{2}\exp(-\frac{\xi^{t}-\xi^{t_{2}}}{\tau_{2}})+E_{2}\Delta \varepsilon_{3}\exp(-\frac{\xi^{t}-\xi^{t_{3}}}{\tau_{2}})\\ \;\;+E_{30}\Delta \varepsilon_{3}+E_{3}\Delta \varepsilon_{3}\exp(-\frac{\xi^{t}-\xi^{t_{3}}}{\tau_{3}}) \end{array}$$

Similarly, at $t_{k}$, there is a strain increment $\Delta \varepsilon_{k}$ in *k* Maxwell elements and *k* spring combinations. The stress after $t_{k}$ is:

(8) $$\delta (t)=\sum_{j=1}^{k}\sum_{i=j}^{k}E_{j0}\Delta \varepsilon_{i}+\sum_{j=1}^{k}\sum_{i=j}^{k}E_{j}\Delta \varepsilon_{i}\exp(-\frac{\xi^{t}-\xi^{t_{i}}}{\tau_{j}})$$

The derivation from Equation (4)–(8) only involves stress analysis for changes in a single Maxwell element within the generalized Maxwell model. For the entire generalized Maxwell model during isothermal curing, the stress at time *t* after time $t_{k}$ is given by:

(9) $$\delta (t)=\sum_{j=1}^{k}\sum_{i=j}^{k}E_{j0}\Delta \varepsilon_{i}+\sum_{m=1}^{n}\sum_{j=1}^{k}\sum_{i=j}^{k}E_{mj}\Delta \varepsilon_{i}\exp(-\frac{\xi^{t}-\xi^{t_{i}}}{\tau_{mj}})$$

where *n* represents the number of initial Maxwell elements in the generalized Maxwell model, and *k* represents the gradual increase in curing reaction, resulting in (*k* − 1) additional Maxwell elements adjacent to each initial Maxwell element.

Equation (9) requires the performance of (*n* × *k*) Maxwell elements and a large number of corresponding parameters, which is quite impractical for real engineering applications. Given the potential for multiple Maxwell models to share the same relaxation time, they can be grouped. In Equation (9), we stipulate that the initial *n* Maxwell models and the newly generated (*k* − 1) Maxwell models are each divided into *n* respective groups, with each group containing Maxwell models sharing the same relaxation time as the initial model. The simplified form of Equation (9) is as follows:

(10) $$\delta (t)=\sum_{j=1}^{k}\sum_{i=j}^{k}E_{j0}\Delta \varepsilon_{i}+\sum_{m=1}^{n}\sum_{j=1}^{k}\sum_{i=j}^{k}E_{mj}\Delta \varepsilon_{i}\exp(-\frac{\xi^{t}-\xi^{t_{i}}}{\tau_{m}})$$

**Figure 3:**
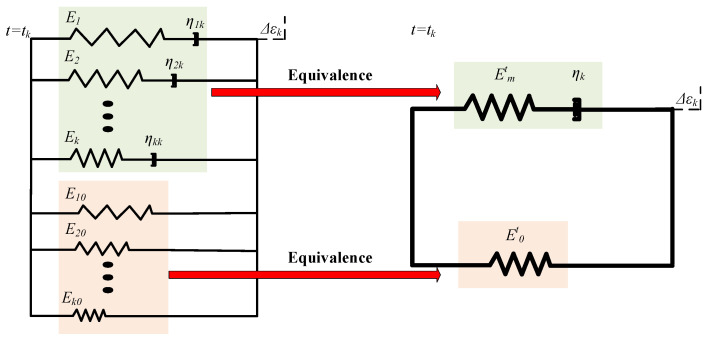
Demonstration of the equivalent evolution of Maxwell elements during the curing process. The green shaded regions denote the Maxwell branches with a common relaxation time, the orange shaded regions denote the equilibrium-spring branches, the ellipses indicate omitted intermediate branches, and the dashed line marks the applied strain increment $\Delta \varepsilon_{k}$.

In the continuous curing process of epoxy resin, the time intervals between $t_{1}$, $t_{2}$ and $t_{3}$ in Figure 2 approach infinitesimally small values, and concurrently, *k* in Equation (10) tends toward infinity. Equation (10) can be reformulated into an integral form:

(11) $$\delta (t)=\int_{0}^{t}E_{0}^{t^{\prime }}\frac{\partial \varepsilon }{\partial t^{\prime }}dt^{\prime }+\sum_{m=1}^{n}\int_{0}^{t}E_{m}^{\xi^{\prime }}\exp(-\frac{\xi^{t}-\xi^{\prime }}{\tau_{m}})\frac{\partial \varepsilon }{\partial \xi^{\prime }}d\xi^{\prime }$$

where $E_{0}^{t}$ and $E_{m}^{\xi^{\prime }}$ represent the increase in the number of parallel springs and Maxwell models, respectively, during curing. This results in the macroscopic fully relaxed and unrelaxed moduli changing with the curing time, as illustrated in Figure 3.

Quantifying the curing process of epoxy resin requires the use of a curing kinetics model, which represents the real-time curing reaction through the degree of cure *α*. Therefore, the changes in the fully relaxed and unrelaxed moduli need to be described by establishing a functional relationship with the degree of cure to reflect the impact of the curing reaction on these moduli. By using the fully relaxed modulus $E_{0}^{\infty }$ and the unrelaxed modulus $E_{m}^{\infty }$ at complete cure as reference values, relationships between $E_{0}^{t}$, $E_{m}^{t}$, and the degree of cure α are established as follows:

(12) $$E_{0}^{t}=\chi (\alpha^{t})E_{0}^{\infty }$$

(13) $$E_{m}^{t}=\psi_{m}(\alpha^{t})E_{m}^{\infty }$$

where $\chi (\alpha^{t})$ and $\psi_{m}(\alpha^{t})$ denote the curing-dependent stiffness factors.

For a specific resin system, the curing-dependent stiffness functions can be identified by combining differential scanning calorimetry (DSC) and dynamic mechanical analysis (DMA). First, isothermal or non-isothermal DSC measurements are used to establish the cure-kinetics model and determine the degree of cure corresponding to each prescribed thermal history. Resin specimens are then partially cured to selected values of α, rapidly cooled to suppress further reaction, and characterized by DMA using frequency sweeps at several temperatures. Temperature- and cure-dependent master curves can subsequently be constructed and fitted using a generalized Maxwell or Prony-series representation. The equilibrium modulus at each cure level provides

(14) $$\chi (\alpha )=\frac{E_{0}(\alpha )}{E_{0}^{\infty }}$$

while the modulus associated with the m-th relaxation branch gives

(15) $$\psi_{m}(\alpha )=\frac{E_{m}(\alpha )}{E_{m}^{\infty }}$$

If a common cure-dependent factor is assumed for all Maxwell branches, a single function $\psi_{m}(\alpha )$ may instead be obtained through a simultaneous fit of the complete relaxation spectrum. The present formulation therefore does not prescribe a universal functional form for χ and $\psi_{m}$; these functions should be calibrated for the resin system of interest. Equation (11) can be changed to:

(16) $$\delta (t)=E_{0}^{\infty }\int_{0}^{t}\chi (\alpha^{t^{\prime }})\frac{\partial \varepsilon }{\partial t^{\prime }}dt^{\prime }+\sum_{m=1}^{n}E_{m}^{\infty }\int_{0}^{t}\psi_{m}(\alpha^{\xi^{\prime }})\exp(-\frac{\xi^{t}-\xi^{\prime }}{\tau_{m}})\frac{\partial \varepsilon }{\partial \xi^{\prime }}d\xi^{\prime }$$

### 2.2. The One-Dimensional Viscoelastic Model for the Non-Isothermal Curing Process

When epoxy resin cures under non-isothermal conditions, temperature variations influence both the spring modulus and the viscosity of the dashpot, as depicted in Figure 1. These temperature-induced changes in the properties of the spring and dashpot are reversible processes. Furthermore, the contributions of thermal gradients and curing reactions to internal stress originate from fundamentally distinct mechanisms. A sudden temperature change affects the stress resulting from strain increments at all previous time points, whereas curing reactions at a given moment influence only the stress generated by strain increments occurring at that specific time point. Stress generated by strain increments at earlier time points remains unaffected by the curing reaction at the final time point. Consistent with the research conducted by Ding et al. [44], Equation (16) is adapted to incorporate the temperature-dependent stiffness parameter:

(17) $$D(T^{t})=\frac{\delta (t,T^{t})}{\delta (t,T_{0})}$$

where $\delta (t,T_{0})$ is the stress at the reference temperature. Then Equation (16) is expressed as:

(18) $$\delta (t)=D(T^{t})\left[E_{0}^{\infty }\int_{0}^{t}\chi (\alpha^{t^{\prime }})\frac{\partial \varepsilon }{\partial t^{\prime }}dt^{\prime }+\sum_{m=1}^{n}E_{m}^{\infty }\int_{0}^{t}\psi_{m}(\alpha^{\xi^{\prime }})\exp(-\frac{\xi^{t}-\xi^{\prime }}{\tau_{m}})\frac{\partial \varepsilon }{\partial \xi^{\prime }}d\xi^{\prime }\right]$$

The curing-dependent stiffness factors and temperature-dependent stiffness parameter were simplified, and Equation (18) is simplified as:

(19) $$\delta (t)=\delta^{t}=D^{t}\left[E_{0}^{\infty }\int_{0}^{t}\chi^{t^{\prime }}\frac{\partial \varepsilon }{\partial t^{\prime }}dt^{\prime }+\sum_{m=1}^{n}E_{m}^{\infty }\int_{0}^{t}\psi_{m}^{\xi^{\prime }}\exp(-\frac{\xi^{t}-\xi^{\prime }}{\tau_{m}})\frac{\partial \varepsilon }{\partial \xi^{\prime }}d\xi^{\prime }\right]$$

where $D^{t}=D(T^{t})$, $\chi^{t}=\chi (\alpha^{t})$ and $\psi_{m}^{t}=\psi_{m}(\alpha^{t})$. By comparing Equations (3) and (19), it is evident that the distinct mechanisms by which temperature and curing reactions influence material stress evolution are effectively reflected in the different integration treatments. The temperature-dependent stiffness factor does not appear in the integration with respect to the time variable t, indicating that within the assumptions of the present formulation, temperature and cure enter the constitutive history through different mechanisms. The parameter D(T) rescales the instantaneous stiffness of all currently active network contributions, while the temperature-dependent shift factor also modifies the reduced-time history and the rate of viscoelastic relaxation. The curing-dependent functions χ(α) and $\psi_{m}(\alpha )$, in contrast, are associated with network contributions activated at different cure states and therefore remain within the history accumulation. This mathematical distinction should not be interpreted as a general thermodynamic statement that temperature effects are universally path-independent. At fixed degree of cure, the formulation reduces to a temperature-dependent viscoelastic model; at fixed temperature, the evolving network contributions retain the cure-history dependence.

### 2.3. The One-Dimensional Material Stress Increment Equations in the Non-Isothermal Curing Process

Equation (19) is essentially an integral-type viscoelastic constitutive equation, and direct application of the integral operation to finite element simulation calculations is challenging. This is because accurate simulation of cure-induced stress in epoxy resin requires a precise definition of the relationship between stress increments and mechanical strain increments within each time increment step. Specifically, the equation relating stress increments to mechanical strain increments needs to be clearly defined for effective simulation. According to Equation (19), the stress at t+Δt is:

(20) $$\begin{array}{l} \delta^{t+\Delta t}=D^{t+\Delta t}\left[E_{0}^{\infty }\int_{0}^{t+\Delta t}\chi^{t^{\prime }}\frac{\partial \varepsilon }{\partial t^{\prime }}dt^{\prime }+\sum_{m=1}^{n}E_{m}^{\infty }\int_{0}^{t+\Delta t}\psi_{m}^{\xi^{\prime }}\exp(-\frac{\xi^{t+\Delta t}-\xi^{\prime }}{\tau_{m}})\frac{\partial \varepsilon }{\partial \xi^{\prime }}d\xi^{\prime }\right]\\ \;\;=D^{t+\Delta t}\left[E_{0}^{\infty }\int_{0}^{t}\chi^{t^{\prime }}\frac{\partial \varepsilon }{\partial t^{\prime }}dt^{\prime }+\sum_{m=1}^{n}E_{m}^{\infty }\int_{0}^{t}\psi_{m}^{\xi^{\prime }}\exp(-\frac{\xi^{t+\Delta t}-\xi^{\prime }}{\tau_{m}})\frac{\partial \varepsilon }{\partial \xi^{\prime }}d\xi^{\prime }\right]+\\ \;\;\;D^{t+\Delta t}\left[E_{0}^{\infty }\int_{t}^{t+\Delta t}\chi^{t^{\prime }}\frac{\partial \varepsilon }{\partial t^{\prime }}dt^{\prime }+\sum_{m=1}^{n}E_{m}^{\infty }\int_{t}^{t+\Delta t}\psi_{m}^{\xi^{\prime }}\exp(-\frac{\xi^{t+\Delta t}-\xi^{\prime }}{\tau_{m}})\frac{\partial \varepsilon }{\partial \xi^{\prime }}d\xi^{\prime }\right] \end{array}$$

Subtracting Equation (19) from Equation (20) yields the stress increment from time t to t+Δt as:

(21) $$\begin{array}{l} \Delta \delta^{t+\Delta t}=\delta^{t+\Delta t}-\delta^{t}\\ \;\;\;=(D^{t+\Delta t}-D^{t})E_{0}^{\infty }\int_{0}^{t}\chi^{t^{\prime }}\frac{\partial \varepsilon }{\partial t^{\prime }}dt^{\prime }+\\ \;\;\;\sum_{m=1}^{n}\;\left[\left(D^{t+\Delta t}\exp(-\frac{\Delta \xi^{t+\Delta t}}{\tau_{m}})-D^{t}\right)\right]E_{m}^{\infty }\int_{0}^{t}\psi_{m}^{\xi^{\prime }}\exp(-\frac{\xi^{t}-\xi^{\prime }}{\tau_{m}})\frac{\partial \varepsilon }{\partial \xi^{\prime }}d\xi^{\prime }+\\ \;\;\;D^{t+\Delta t}E_{0}^{\infty }\int_{t}^{t+\Delta t}\chi^{t^{\prime }}\frac{\partial \varepsilon }{\partial t^{\prime }}dt^{\prime }+\\ \;\;\;D^{t+\Delta t}\sum_{m=1}^{n}E_{m}^{\infty }\int_{t}^{t+\Delta t}\psi_{m}^{\xi^{\prime }}\exp(-\frac{\xi^{t+\Delta t}-\xi^{\prime }}{\tau_{m}})\frac{\partial \varepsilon }{\partial \xi^{\prime }}d\xi^{\prime } \end{array}$$

where $\Delta \xi^{t+\Delta t}$ is the increment of reduced time, and $\Delta \xi^{t+\Delta t}=\xi^{t+\Delta t}-\xi^{t}$. It can also be written as:

(22) $$\Delta \xi^{t+\Delta t}=\int_{t}^{t+\Delta t}\frac{ds}{a_{\left(T,\alpha \right)}^{s}}=\int_{t}^{t+\Delta t}\frac{ds}{a^{s}}$$

The stress increment given by Equation (21) still involves integration and cannot be directly applied to finite element analysis. It is therefore necessary to recast this integral operation into a form compatible with the finite element computational framework. In this context, the interval Δt from t to t+Δt is considered as a discrete step. Within each time increment, the finite element analysis framework maintains constant values for the curing-dependent stiffness factors, the temperature-dependent stiffness parameters, temperature, the degree of curing, and the shift factor. Thus, $\Delta \xi^{t+\Delta t}$ can be simplified as:

(23) $$\Delta \xi^{t+\Delta t}=\frac{\Delta t^{t+\Delta t}}{a^{t+\Delta t}}$$

where $\Delta t^{t+\Delta t}$ is the time increment from t to t+Δt. At the same time, the strain increment $\Delta \varepsilon^{t+\Delta t}$ from t to t+Δt changes linearly with the reduction time $\xi^{\prime }$:

(24) $$\frac{\partial \varepsilon }{\partial \xi^{\prime }}=\frac{\Delta \varepsilon^{t+\Delta t}}{\Delta \xi^{t+\Delta t}}=a^{t+\Delta t}\frac{\Delta \varepsilon^{t+\Delta t}}{\Delta t^{t+\Delta t}}$$

Hence, the integral calculation in Equation (21) undergoes the following transformation:

(25) $$\begin{array}{l} \int_{0}^{t}\chi^{t^{\prime }}\frac{\partial \varepsilon }{\partial t^{\prime }}dt^{\prime }=\int_{0}^{t-\Delta t}\chi^{t^{\prime }}\frac{\partial \varepsilon }{\partial t^{\prime }}dt^{\prime }+\int_{t-\Delta t}^{t}\chi^{t^{\prime }}\frac{\partial \varepsilon }{\partial t^{\prime }}dt^{\prime }\\ \;\;\;\;=\int_{0}^{t-\Delta t}\chi^{t^{\prime }}\frac{\partial \varepsilon }{\partial t^{\prime }}dt^{\prime }+\chi^{t}\Delta \varepsilon^{t} \end{array}$$

(26) $$\begin{array}{l} \int_{0}^{t}\psi_{m}^{\xi^{\prime }}\exp(-\frac{\xi^{t}-\xi^{\prime }}{\tau_{m}})\frac{\partial \varepsilon }{\partial \xi^{\prime }}d\xi^{\prime }=\int_{0}^{t-\Delta t}\psi_{m}^{\xi^{\prime }}\exp(-\frac{\xi^{t}-\xi^{\prime }}{\tau_{m}})\frac{\partial \varepsilon }{\partial \xi^{\prime }}d\xi^{\prime }+\int_{t-\Delta t}^{t}\psi_{m}^{\xi^{\prime }}\exp(-\frac{\xi^{t}-\xi^{\prime }}{\tau_{m}})\frac{\partial \varepsilon }{\partial \xi^{\prime }}d\xi^{\prime }\\ \;\;\;\;\;\;\;\;\;\;=\exp(-\frac{\Delta \xi^{t}}{\tau_{m}})\int_{0}^{t-\Delta t}\psi_{m}^{\xi^{\prime }}\exp(-\frac{\xi^{t-\Delta t}-\xi^{\prime }}{\tau_{m}})\frac{\partial \varepsilon }{\partial \xi^{\prime }}d\xi^{\prime }+\psi_{m}^{t}\tau_{m}a^{t}\frac{\Delta \varepsilon^{t}}{\Delta t^{t}}\left[1-\exp(-\frac{\Delta \xi^{t}}{\tau_{m}})\right] \end{array}$$

(27) $$\int_{t}^{t+\Delta t}\chi^{t^{\prime }}\frac{\partial \varepsilon }{\partial t^{\prime }}dt^{\prime }=\chi^{t+\Delta t}\Delta \varepsilon^{t+\Delta t}$$

(28) $$\int_{t}^{t+\Delta t}\psi_{m}^{\xi^{\prime }}\exp(-\frac{\xi^{t+\Delta t}-\xi^{\prime }}{\tau_{m}})\frac{\partial \varepsilon }{\partial \xi^{\prime }}d\xi^{\prime }=\psi_{m}^{t+\Delta t}\tau_{m}a^{t+\Delta t}\frac{\Delta \varepsilon^{t+\Delta t}}{\Delta t^{t+\Delta t}}\left[1-\exp(-\frac{\Delta \xi^{t+\Delta t}}{\tau_{m}})\right]$$

In summary, the stress increment from t to t+Δt is:

(29) $$\begin{array}{l} \Delta \delta^{t+\Delta t}=(D^{t+\Delta t}-D^{t})E_{0}^{\infty }P^{t}+\\ \;\;\;\;\sum_{m=1}^{n}\;\left[\left(D^{t+\Delta t}\exp(-\frac{\Delta \xi^{t+\Delta t}}{\tau_{m}})-D^{t}\right)\right]E_{m}^{\infty }Q_{m}^{t}+\\ \;\;\;\;D^{t+\Delta t}E_{0}^{\infty }\chi^{t+\Delta t}\Delta \varepsilon^{t+\Delta t}+\\ \;\;\;\;D^{t+\Delta t}\sum_{m=1}^{n}E_{m}^{\infty }\psi_{m}^{t+\Delta t}\tau_{m}a^{t+\Delta t}\frac{\Delta \varepsilon^{t+\Delta t}}{\Delta t^{t+\Delta t}}\left[1-\exp(-\frac{\Delta \xi^{t+\Delta t}}{\tau_{m}})\right] \end{array}$$

where $P^{t}$ and $Q_{m}^{t}$ are:

(30) $$P^{t}=P^{t-\Delta t}+\chi^{t}\Delta \varepsilon^{t}$$

(31) $$Q_{m}^{t}=\exp(-\frac{\Delta \xi^{t}}{\tau_{k}})Q_{m}^{t-\Delta t}+\psi_{m}^{t}\tau_{m}a^{t}\frac{\Delta \varepsilon^{t}}{\Delta t^{t}}\left[1-\exp(-\frac{\Delta \xi^{t}}{\tau_{m}})\right]$$

**Figure 4:**
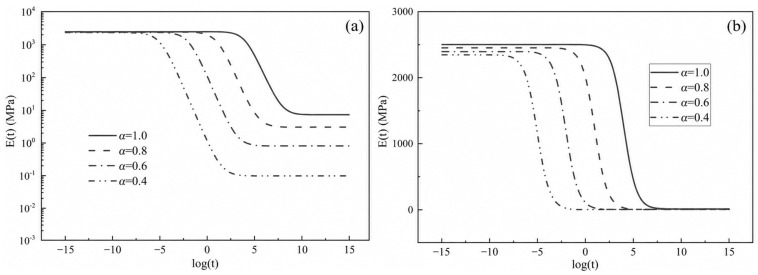
Variation in the relaxation modulus with degree of cure during curing: (**a**) relaxation modulus plotted on a logarithmic scale; (**b**) relaxation modulus plotted on a linear scale.

The initial values of $P^{t}$ and $Q_{m}^{t}$ are 0. For the stress increment in Equation (29), the number *n* represents the number of curing-dependent stiffness factors ψ, indicating that each of the *n* initial Maxwell units has a distinct curing-dependent stiffness factor. This suggests that the impact of the curing reaction on each Maxwell unit is not proportional or synchronized. However, many engineers and researchers assume that the generalized Maxwell model’s *n* initial Maxwell units share the same curing-dependent stiffness factor, based on practical applications. When all curing-dependent stiffness factors ψ are considered equal, the variation in the relaxation modulus *E*(*t*) with the degree of cure can be displayed in Figure 4. Notably, the resulting E(t) curves for different degrees of cure exhibit parallel trends in the significant decline region. Therefore, when all initial Maxwell units have the same curing-dependent stiffness factor, Equation (19) can be rewritten in a more compact form as Equation (32).

(32) $$\delta (t)=\delta^{t}=D^{t}\left[E_{0}^{\infty }\int_{0}^{t}\chi^{t^{\prime }}\frac{\partial \varepsilon }{\partial t^{\prime }}dt^{\prime }+\bar{E}\sum_{m=1}^{n}W_{m}\int_{0}^{t}\psi^{\xi^{\prime }}\exp(-\frac{\xi^{t}-\xi^{\prime }}{\tau_{m}})\frac{\partial \varepsilon }{\partial \xi^{\prime }}d\xi^{\prime }\right]$$

where $\bar{E}=\sum_{m=1}^{n}E_{m}^{\infty }$; $W_{m}$ is the weight factor, and $W_{m}=E_{m}^{\infty }/\bar{E}$. The macroscopically curing-dependent one-dimensional relaxation modulus is:

(33) $$E^{t}=D(T)\left[E_{0}^{\infty }\chi^{t}+\bar{E}\sum_{m=1}^{n}W_{m}\psi^{t}\exp(-\frac{t}{a_{\left(\alpha ,T\right)}\tau_{m}})\right]$$

Then the one-dimensional stress increment in Equation (29) ultimately transforms to:

(34) $$\begin{array}{l} \Delta \delta^{t+\Delta t}=(D^{t+\Delta t}-D^{t})E_{0}^{\infty }P^{t}+\\ \;\;\;\bar{E}\sum_{m=1}^{n}\;\left[\left(D^{t+\Delta t}\exp(-\frac{\Delta \xi^{t+\Delta t}}{\tau_{m}})-D^{t}\right)\right]W_{m}Q_{m}^{t}+\\ \;\;\;D^{t+\Delta t}E_{0}^{\infty }\chi^{t+\Delta t}\Delta \varepsilon^{t+\Delta t}+\\ \;\;\;D^{t+\Delta t}\psi^{t+\Delta t}\bar{E}a^{t+\Delta t}\frac{\Delta \varepsilon^{t+\Delta t}}{\Delta t^{t+\Delta t}}\sum_{m=1}^{n}W_{m}\tau_{m}\left[1-\exp(-\frac{\Delta \xi^{t+\Delta t}}{\tau_{m}})\right] \end{array}$$

where:

(35) $$P^{t}=P^{t-\Delta t}+\chi^{t}\Delta \varepsilon^{t}$$

(36) $$Q_{m}^{t}=\exp(-\frac{\Delta \xi^{t}}{\tau_{k}})Q_{m}^{t-\Delta t}+\psi^{t}\tau_{m}a^{t}\frac{\Delta \varepsilon^{t}}{\Delta t^{t}}\left[1-\exp(-\frac{\Delta \xi^{t}}{\tau_{m}})\right]$$

In contrast to Equation (29), Equation (34) is more concise and requires fewer material parameters, thus lending itself more readily to practical engineering applications. However, it is important to note that Equation (34) is more idealized, whereas Equation (29) provides a more accurate description of viscoelastic response.

### 2.4. The Stress Increment Equations of Three-Dimensional Epoxy Resin Material During the Non-Isothermal Curing Process

The three-dimensional integral viscoelastic constitutive equation is defined as:

(37) $$\delta_{i}^{t}=\int_{0}^{t}C_{ij}^{t-t^{\prime }}\frac{\partial \varepsilon_{j}}{\partial t^{\prime }}dt^{\prime }$$

where $C_{ij}^{t}$ is the stiffness matrix. Epoxy resin is an isotropic material. To simplify the stiffness matrix and facilitate the computation of stress increments, the shear relaxation modulus $G^{t}$ and bulk relaxation modulus $K^{t}$ are used to represent the stiffness matrix. The stiffness matrix can be expanded as:

(38) $$C^{t}=\left\{\begin{array}{cccccc} K^{t}+\frac{4}{3}G^{t} & K^{t}-\frac{2}{3}G^{t} & K^{t}-\frac{2}{3}G^{t} & 0 & 0 & 0\\ K^{t}-\frac{2}{3}G^{t} & K^{t}+\frac{4}{3}G^{t} & K^{t}-\frac{2}{3}G^{t} & 0 & 0 & 0\\ K^{t}-\frac{2}{3}G^{t} & K^{t}-\frac{2}{3}G^{t} & K^{t}+\frac{4}{3}G^{t} & 0 & 0 & 0\\ 0 & 0 & 0 & G^{t} & 0 & 0\\ 0 & 0 & 0 & 0 & G^{t} & 0\\ 0 & 0 & 0 & 0 & 0 & G^{t} \end{array}\right\}$$

Referring to Equation (33), the shear relaxation modulus $G^{t}$ and the bulk relaxation modulus $K^{t}$ are expressed as:

(39) $$K^{t}=D_{K}(T)\left[\chi_{K}^{t}K_{0}^{\infty }+\bar{K}\sum_{m=1}^{n1}W_{Km}\psi_{K}^{t}\exp(-\frac{t}{a_{K(T,\alpha )}\tau_{Km}})\right]$$

(40) $$G^{t}=D_{G}(T)\left[\chi_{G}^{t}G_{0}^{\infty }+\bar{G}\sum_{m=1}^{n2}W_{Gm}\psi_{G}^{t}\exp(-\frac{t}{a_{G(T,\alpha )}\tau_{Gm}})\right]$$

Then Equation (37) can be decomposed into:

(41) $$\begin{array}{l} \delta_{i}^{t}=\int_{0}^{t}\left(A_{ij}K^{t-t^{\prime }}+B_{ij}G^{t-t^{\prime }}\right)\frac{\partial \varepsilon_{j}}{\partial t^{\prime }}dt^{\prime }\\ \;=A_{ij}\int_{0}^{t}K^{t-t^{\prime }}\frac{\partial \varepsilon_{j}}{\partial t^{\prime }}dt^{\prime }+B_{ij}\int_{0}^{t}G^{t-t^{\prime }}\frac{\partial \varepsilon_{j}}{\partial t^{\prime }}dt^{\prime } \end{array}$$

where $A_{ij}$ and $B_{ij}$ are given by:

(42) $$A=\left\{\begin{array}{cccccc} 1 & 1 & 1 & 0 & 0 & 0\\ 1 & 1 & 1 & 0 & 0 & 0\\ 1 & 1 & 1 & 0 & 0 & 0\\ 0 & 0 & 0 & 0 & 0 & 0\\ 0 & 0 & 0 & 0 & 0 & 0\\ 0 & 0 & 0 & 0 & 0 & 0 \end{array}\right\}$$

(43) $$B=\left\{\begin{array}{cccccc} \frac{4}{3} & \frac{-2}{3} & \frac{-2}{3} & 0 & 0 & 0\\ \frac{-2}{3} & \frac{4}{3} & \frac{-2}{3} & 0 & 0 & 0\\ \frac{-2}{3} & \frac{-2}{3} & \frac{4}{3} & 0 & 0 & 0\\ 0 & 0 & 0 & 1 & 0 & 0\\ 0 & 0 & 0 & 0 & 1 & 0\\ 0 & 0 & 0 & 0 & 0 & 1 \end{array}\right\}$$

Based on the derivation of the one-dimensional stress increment equations, the corresponding equations for epoxy resin during curing can be derived from Equation (41).

(44) $$\Delta \delta_{i}^{t+\Delta t}=A_{ij}\Delta \delta_{Ki}^{t+\Delta t}+B_{ij}\Delta \delta_{Gi}^{t+\Delta t}$$

where $\Delta \delta_{Ki}^{t+\Delta t}$ and $\Delta \delta_{Gi}^{t+\Delta t}$ are represented below:

(45) $$\begin{array}{l} \Delta \delta_{Ki}^{t+\Delta t}=(D_{K}^{t+\Delta t}-D_{K}^{t})K_{0}^{\infty }P_{K}^{t}+\\ \;\;\;\bar{K}\sum_{m=1}^{n1}\left[D_{K}^{t+\Delta t}\exp(-\frac{\Delta \xi_{K}^{t+\Delta t}}{\tau_{Km}})-D_{K}^{t}\right]W_{Km}Q_{Km}^{t}+\\ \;\;\;D_{K}^{t+\Delta t}K_{0}^{\infty }\chi_{K}^{t+\Delta t}\Delta \varepsilon_{i}^{t+\Delta t}+\\ \;\;\;D_{K}^{t+\Delta t}\bar{K}\sum_{m=1}^{n1}W_{Km}\psi_{K}^{t+\Delta t}\tau_{Km}a_{K}^{t+\Delta t}\frac{\Delta \varepsilon^{t+\Delta t}}{\Delta t^{t+\Delta t}}\left[1-\exp(-\frac{\Delta \xi_{K}^{t+\Delta t}}{\tau_{Km}})\right] \end{array}$$

(46) $$\begin{array}{l} \Delta \delta_{Gi}^{t+\Delta t}=(D_{G}^{t+\Delta t}-D_{G}^{t})K_{0}^{\infty }P_{G}^{t}+\\ \;\;\;\bar{G}\sum_{m=1}^{n2}\left[D_{G}^{t+\Delta t}\exp(-\frac{\Delta \xi_{G}^{t+\Delta t}}{\tau_{Gm}})-D_{G}^{t}\right]W_{Gm}Q_{Gm}^{t}+\\ \;\;\;D_{G}^{t+\Delta t}G_{0}^{\infty }\chi_{G}^{t+\Delta t}\Delta \varepsilon_{i}^{t+\Delta t}+\\ \;\;\;D_{G}^{t+\Delta t}\bar{G}\sum_{m=1}^{n2}W_{Gm}\psi_{G}^{t+\Delta t}\tau_{Gm}a_{G}^{t+\Delta t}\frac{\Delta \varepsilon^{t+\Delta t}}{\Delta t^{t+\Delta t}}\left[1-\exp(-\frac{\Delta \xi_{G}^{t+\Delta t}}{\tau_{Gm}})\right] \end{array}$$

where:

(47) $$P_{K}^{t}=P_{K}^{t-\Delta t}+\chi_{K}^{t}\Delta \varepsilon_{i}^{t}$$

(48) $$Q_{Km}^{t}=\exp(-\frac{\Delta \xi_{K}^{t}}{\tau_{Km}})Q_{Km}^{t-\Delta t}+\psi_{K}^{t}\tau_{Km}a^{t}\frac{\Delta \varepsilon_{i}^{t}}{\Delta t^{t}}\left[1-\exp(-\frac{\Delta \xi_{K}^{t}}{\tau_{Km}})\right]$$

(49) $$P_{G}^{t}=P_{G}^{t-\Delta t}+\chi_{G}^{t}\Delta \varepsilon_{i}^{t}$$

(50) $$Q_{Gm}^{t}=\exp(-\frac{\Delta \xi_{G}^{t}}{\tau_{Gm}})Q_{Gm}^{t-\Delta t}+\psi_{G}^{t}\tau_{Gm}a_{G}^{t}\frac{\Delta \varepsilon_{i}^{t}}{\Delta t^{t}}\left[1-\exp(-\frac{\Delta \xi_{G}^{t}}{\tau_{Gm}})\right]$$

For fully cured epoxy resin, the value of curing-dependent stiffness factors in the above equations is 1, and its internal stress increment under external action is simplified as:

(51) $$\begin{array}{l} \Delta \delta_{Ki}^{t+\Delta t}=(D_{K}^{t+\Delta t}-D_{K}^{t})K_{0}^{\infty }\varepsilon_{j}^{t}+\\ \;\;\;\bar{K}\;\sum_{m=1}^{n1}\left[D_{K}^{t+\Delta t}\exp(-\frac{\Delta \xi_{K}^{t+\Delta t}}{\tau_{Km}})-D_{K}^{t}\right]W_{Km}Q_{Km}^{t}+\\ \;\;\;D_{K}^{t+\Delta t}K_{0}^{\infty }\Delta \varepsilon_{i}^{t+\Delta t}+\\ \;\;\;D_{K}^{t+\Delta t}\bar{K}\sum_{m=1}^{n1}W_{Km}\tau_{Km}a_{K}^{t+\Delta t}\frac{\Delta \varepsilon_{i}^{t+\Delta t}}{\Delta t^{t+\Delta t}}\left[1-\exp(-\frac{\Delta \xi_{K}^{t+\Delta t}}{\tau_{Km}})\right] \end{array}$$

(52) $$\begin{array}{l} \Delta \delta_{Gi}^{t+\Delta t}=(D_{G}^{t+\Delta t}-D_{G}^{t})K_{0}^{\infty }\varepsilon_{j}^{t}+\\ \;\;\;\bar{G}\sum_{m=1}^{n2}\left[D_{G}^{t+\Delta t}\exp(-\frac{\Delta \xi_{G}^{t+\Delta t}}{\tau_{Gm}})-D_{G}^{t}\right]W_{Gm}Q_{Gm}^{t}+\\ \;\;\;D_{G}^{t+\Delta t}G_{0}^{\infty }\Delta \varepsilon_{i}^{t+\Delta t}+\\ \;\;\;D_{G}^{t+\Delta t}\bar{G}\sum_{m=1}^{n2}W_{Gm}\tau_{Gm}a_{G}^{t+\Delta t}\frac{\Delta \varepsilon_{i}^{t+\Delta t}}{\Delta t^{t+\Delta t}}\left[1-\exp(-\frac{\Delta \xi_{G}^{t+\Delta t}}{\tau_{Gm}})\right] \end{array}$$

where:

(53) $$Q_{Km}^{t}=\exp(-\frac{\Delta \xi_{K}^{t}}{\tau_{Km}})Q_{Km}^{t-\Delta t}+\tau_{Km}a^{t}\frac{\Delta \varepsilon^{t}}{\Delta t^{t}}\left[1-\exp(-\frac{\Delta \xi_{K}^{t}}{\tau_{Km}})\right]$$

(54) $$Q_{Gm}^{t}=\exp(-\frac{\Delta \xi_{G}^{t}}{\tau_{Gm}})Q_{Gm}^{t-\Delta t}+\tau_{Gm}a_{G}^{t}\frac{\Delta \varepsilon_{i}^{t}}{\Delta t^{t}}\left[1-\exp(-\frac{\Delta \xi_{G}^{t}}{\tau_{Gm}})\right]$$

### 2.5. Formal Tensorial Extension to Anisotropic Thermosetting Materials

Epoxy resin is widely used as a common matrix phase in composite materials, necessitating the transfer of cure-induced stress increment formulations to the composite materials domain to advance this field. On a macroscopic scale, resin-based composites are anisotropic during curing. Unlike isotropic materials, for which two independent material parameters can represent the stiffness matrix, the stiffness matrix in the three-dimensional integral-type viscoelastic constitutive equation for anisotropic materials in Equation (37) is given by:

(55) $$C_{ij}^{t}=D(T)\left[\chi^{t}C_{ij}^{\infty }+{\bar{C}}_{ij}\sum_{m=1}^{n}W_{m}\psi^{t}\exp(-\frac{t}{a_{\left(T,\alpha \right)}\tau_{m}})\right]$$

Thus, for anisotropic materials, Equation (37) can be expanded to:

(56) $$\delta_{i}^{t}=D^{t}\left[C_{ij}^{\infty }\int_{0}^{t}\chi^{t^{\prime }}\frac{\partial \varepsilon_{j}}{\partial t^{\prime }}dt^{\prime }+{\bar{C}}_{ij}\sum_{m=1}^{n}W_{m}\int_{0}^{t}\psi^{\xi^{\prime }}\exp(-\frac{\xi^{t}-\xi^{\prime }}{\tau_{m}})\frac{\partial \varepsilon_{j}}{\partial \xi^{\prime }}d\xi^{\prime }\right]$$

Following a similar derivation process as for isotropic materials, the stress increment in Equation (56) can be determined as:

(57) $$\begin{array}{l} \Delta \delta_{i}^{t+\Delta t}=(D^{t+\Delta t}-D^{t})C_{ij}^{\infty }P_{j}^{t}+\\ \;\;\;{\bar{C}}_{ij}\sum_{m=1}^{n}\;\left[\left(D^{t+\Delta t}\exp(-\frac{\Delta \xi^{t+\Delta t}}{\tau_{m}})-D^{t}\right)\right]W_{m}Q_{m}^{t}+\\ \;\;\;D^{t+\Delta t}C_{ij}^{\infty }\chi^{t+\Delta t}\Delta \varepsilon_{j}^{t+\Delta t}+\\ \;\;\;D^{t+\Delta t}\psi^{t+\Delta t}{\bar{C}}_{ij}a^{t+\Delta t}\frac{\Delta \varepsilon^{t+\Delta t}}{\Delta t^{t+\Delta t}}\sum_{m=1}^{n}W_{m}\tau_{m}\left[1-\exp(-\frac{\Delta \xi^{t+\Delta t}}{\tau_{m}})\right] \end{array}$$

where:

(58) $$P_{j}^{t}=P_{j}^{t-\Delta t}+\chi^{t}\Delta \varepsilon_{j}^{t}$$

(59) $$Q_{mj}^{t}=\exp(-\frac{\Delta \xi^{t}}{\tau_{k}})Q_{mj}^{t-\Delta t}+\psi^{t}\tau_{m}a^{t}\frac{\Delta \varepsilon_{j}^{t}}{\Delta t^{t}}\left[1-\exp(-\frac{\Delta \xi^{t}}{\tau_{m}})\right]$$

Although the derivation assumes identical temperature, curing, weight, displacement, and relaxation factors across all stiffness-matrix components, a distinct notational convention is required for Equations (55)–(59) when these parameters vary by component. Equations (55)–(59) provide only a formal tensorial extension under the simplifying assumption that the temperature, cure, relaxation, and weighting functions are common to the relevant stiffness components. Application to an actual thermosetting composite would require direction-dependent cure shrinkage, orthotropic relaxation spectra, and independently calibrated component-wise stiffness functions. These aspects, together with numerical verification of the anisotropic formulation, are outside the scope of the present study.

## 3. Constitutive and UMAT Verification Using Idealized Parameters

Spatially uniform temperature and degree-of-cure histories are prescribed in the verification cases so that every integration point follows the same constitutive loading path. This permits direct pointwise comparison between the UMAT result and the independently evaluated stress-increment equation. The uniform-field assumption is therefore a verification design choice and should not be interpreted as a representation of an actual industrial curing process.

The viscoelastic constitutive model for epoxy resin can be implemented using the UMAT user subroutine in the finite element package ABAQUS, enabling the simulation of both the curing stage and subsequent post-cure behavior, with the three-dimensional variable-temperature stress-increment equations integrated into the framework. After establishing the resin material model and assigning appropriate boundary conditions within ABAQUS, static analyses are conducted by invoking the UMAT subroutine. The numerical implementation of the derived stress-increment equations was verified by comparing the UMAT results with stresses obtained through direct evaluation of the constitutive equations. The two calculations were performed using identical material parameters, temperature histories, degree-of-cure histories, strain histories, and time increments. Therefore, the comparison evaluates the consistency of the stress-update algorithm rather than the physical predictive capability of the constitutive model.

To quantify the numerical differences, the maximum absolute error, final-time absolute error, and root-mean-square deviation were calculated as

(60) $$e_{\max}=\underset{1\leq i\leq N}{\max}\left|\sigma_{i}^{UMAT}-\sigma_{i}^{direct}\right|$$

(61) $$e_{final}=\left|\sigma_{N}^{UMAT}-\sigma_{N}^{direct}\right|$$

and

(62) $$e_{RMS}=\sqrt{\frac{1}{N}\sum_{i=1}^{N}{\left(\sigma_{i}^{UMAT}-\sigma_{i}^{direct}\right)}^{2}}$$

where N is the number of output points, while $\sigma_{i}^{UMAT}$ and $\sigma_{i}^{direct}$ denote the stresses obtained from the UMAT calculation and direct constitutive evaluation, respectively.

### 3.1. Illustrative Example 1

**Figure 5:**
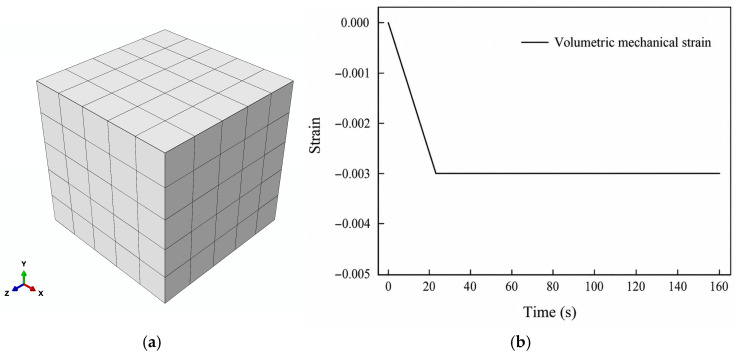
(**a**) The cubic finite element model. (**b**) The variation in model volume strain over time.

**Figure 6:**
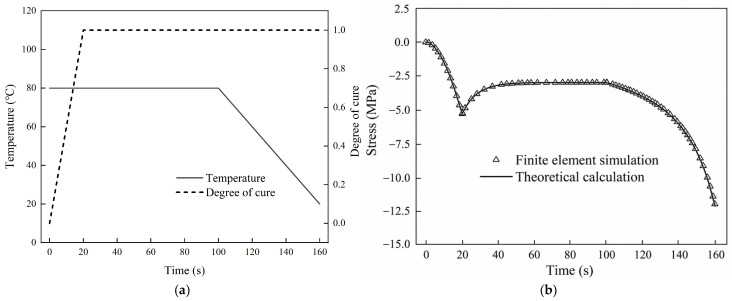
(**a**) The variation in temperature and degree of cure over time. (**b**) The comparison between the simulated stress and the theoretical stress of the resin material model along the *X*-axis direction.

A solid model of the resin, with an edge length of 50 mm, was constructed as shown in Figure 5a. Material properties were assigned via the UMAT user subroutine, and simplified parameters from Table 1 were adopted to facilitate comparison between simulation and theory. The values in Table 1 are idealized parameters selected to isolate the contributions of cure-dependent stiffness evolution, temperature-dependent stiffness, and viscoelastic relaxation. They are not intended to represent a specific commercial epoxy resin. Under centripetal compression applied to the six faces, volumetric mechanical strain develops within the cube and evolves with time, as illustrated in Figure 5b. Temperature and degree of cure are uniformly distributed across the model, with their temporal variations presented in Figure 6a. The mesh was configured with C3D8I elements, and an implicit solver was employed to analyze the cure-induced stress. Concurrently, based on the material constants in Table 1, the theoretical stress along the X-axis of the model in Figure 5 was computed as a function of time. The UMAT result and the stress obtained by direct constitutive evaluation are compared in Figure 6b. As summarized in Table 2, the maximum absolute error is 0.165 MPa, the final-time absolute error is 0.165 MPa, and the RMS deviation is 0.054 MPa. These small differences demonstrate that the UMAT stress-update procedure consistently reproduces the direct evaluation of the derived stress-increment equation for Verification Case 1.

During the initial 0–20 s interval, the volumetric mechanical strain and the degree of cure increase simultaneously. Consequently, the magnitude of the compressive stress increases, and the stress reaches −5.27 MPa at t = 20 s, because the increase in load-carrying stiffness exceeds the simultaneous viscoelastic relaxation. Between 20 and 100 s, both the prescribed strain and degree of cure remain constant, and the stress decreases from −5.27 MPa to −2.99 MPa as a result of viscoelastic relaxation. During the subsequent cooling interval from 100 to 160 s, the mechanical strain remains unchanged, while the decrease in temperature increases the temperature-dependent stiffness parameter. The magnitude of the compressive stress therefore increases, with the stress changing from −2.99 MPa to a final value of −12.0 MPa. These three stages separately illustrate the combined effects of mechanical loading and stiffness buildup, viscoelastic relaxation, and temperature-dependent stiffness variation under the prescribed constitutive assumptions.

### 3.2. Illustrative Example 2

**Figure 7:**
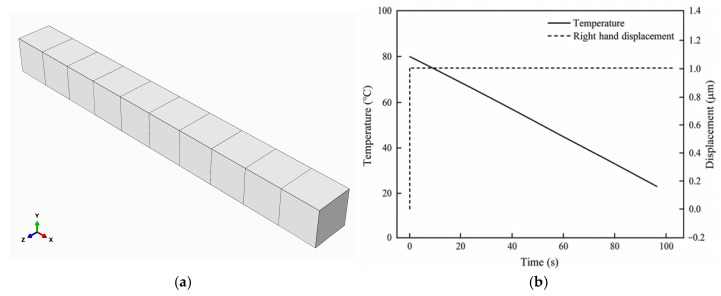
(**a**) The cuboid finite element model. (**b**) Right-side displacement and model temperature change over time.

**Figure 8:**
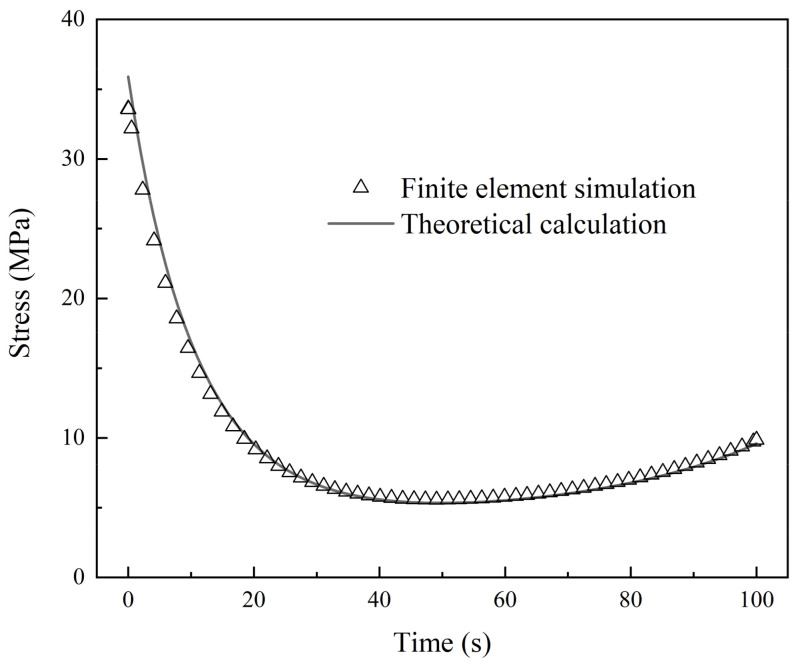
Comparison of simulated stress and theoretical stress of the fully cured resin material model along the *X*-axis.

A fully cured finite element model of the resin material, with an edge length of 100 mm, was established as depicted in Figure 7a. Material properties, supplied via the UMAT user subroutine and summarized in Table 1, correspond to a fully cured resin with a degree of cure equal to unity. Figure 7b presents the prescribed temperature history and the displacement applied to the right end face over time. The temperature varies linearly, while the left end face remains fixed. At the initial instant, the right end face is assigned a positive *X*-direction displacement. The mesh was configured with C3D8I elements, and implicit analysis was employed to simulate the stress relaxation response. Simultaneously, theoretical stress histories along the *X*-axis were computed for comparison with the simulation results. Figure 8 compares the UMAT stress history with the stress obtained by direct constitutive evaluation. The quantitative errors are also listed in Table 2. The maximum absolute error is 2.299 MPa, the final-time absolute error is 0.262 MPa, and the RMS deviation is 0.597 MPa. These results verify the numerical consistency of the UMAT implementation for the fully cured, non-isothermal relaxation case.

At the beginning of the analysis, the prescribed displacement produces an instantaneous stress of approximately 35 MPa. From 0 to 50 s, the stress decreases from approximately 35 MPa to 5.33 MPa because of viscoelastic relaxation, while the influence of the temperature-dependent stiffness variation remains relatively limited during this interval. From 50 to 100 s, the prescribed temperature decreases from 52.5 °C to 25 °C. Under the adopted constitutive assumptions, the corresponding increase in the temperature-dependent stiffness parameter causes the stress to rise from 5.33 MPa to a final value of 9.6 MPa. This result illustrates the interaction between viscoelastic relaxation and temperature-dependent stiffness in the fully cured verification case; it should not be interpreted as a universal statement that the material response is governed solely by temperature.

As shown in Table 2, the errors remain small throughout both loading histories. Verification Case 1 examines the combined effects of mechanical strain, cure-dependent stiffness evolution, viscoelastic relaxation, and temperature-dependent stiffness, whereas Verification Case 2 focuses on the relaxation and temperature-dependent response of the fully cured resin. The agreement obtained in both cases demonstrates that the derived stress-increment equations have been consistently implemented in the UMAT. Nevertheless, because both reference solutions are calculated from the same constitutive equations, these comparisons constitute numerical verification rather than independent physical validation.

## 4. Investigation of the Influence of Curing-Dependent Stiffness Factors

### 4.1. Encapsulation Model and Quantitative Stress Analysis

**Figure 9:**
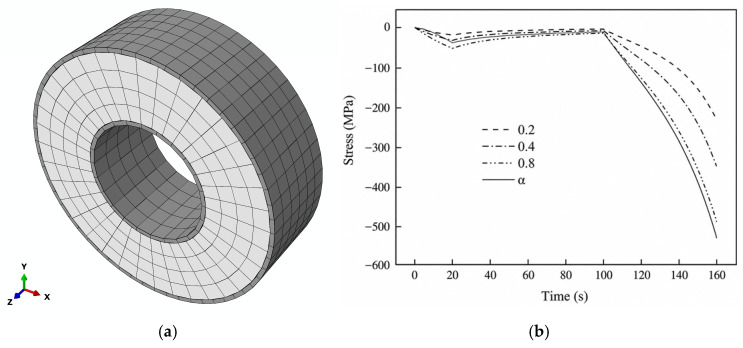
(**a**) Resin package structure model. (**b**) The variation in circumferential stress of the outer steel layer.

Accordingly, the following encapsulation model is used as an illustrative sensitivity case rather than as a material-specific prediction. Figure 9a depicts the package structure of the epoxy resin. This ring-shaped model consists of a resin middle layer sandwiched between inner and outer steel layers, each 1 mm thick, with an outer diameter of 60 mm, an inner diameter of 28 mm, and a width of 20 mm. The steel–resin interfaces were modeled using perfect tie constraints. Accordingly, displacement compatibility was enforced across each interface, and interfacial sliding, opening, and damage were not considered. This assumption was adopted to isolate the influence of the resin constitutive response.

A mesh-convergence study was performed using 612, 1218, and 1948 elements, corresponding to characteristic element sizes of 6.0, 4.0, and 2.5 mm. The resulting local cure-ramp peak circumferential stresses were −29.28, −37.62, and −36.73 MPa. Since the difference between the medium and fine meshes was 2.42%, the medium mesh was used in the subsequent calculations.

The viscoelastic properties of the epoxy resin are consistent with those listed in Table 1. Both temperature and the degree of cure are uniformly distributed throughout the model and follow the same trends shown in Figure 6a. The thermal expansion coefficients for the resin (100 × 10^−6^/°C) and steel (11.7 × 10^−6^/°C), along with the Young’s modulus (206 GPa) and Poisson’s ratio (0.3) of steel, are specified. Curing shrinkage of the resin induces a time-varying volumetric strain that corresponds to the curve in Figure 5b. The model is meshed with C3D8I elements and analyzed using an implicit static procedure.

Figure 9b presents the temporal evolution of circumferential stress in the outer steel layer under various conditions. Here, *α* denotes the influence of the curing-dependent stiffness factor, which varies with the degree of cure during the actual simulation; the fixed values 0.2, 0.4, and 0.8 correspond to constant stiffness factors at degrees of cure 0.2, 0.4, and 0.8, respectively. As illustrated in Figure 9b, these stiffness influence factors exert a substantial effect on the package stress during both the initial cure-ramp stage and the subsequent cooling phase. After 100 s, the rapid increase in the magnitude of the compressive circumferential stress is primarily caused by the mismatch in thermal contraction between the resin and steel during cooling, while the curing-dependent stiffness-factor assumption controls the magnitude of the resulting response.

**Figure 10:**
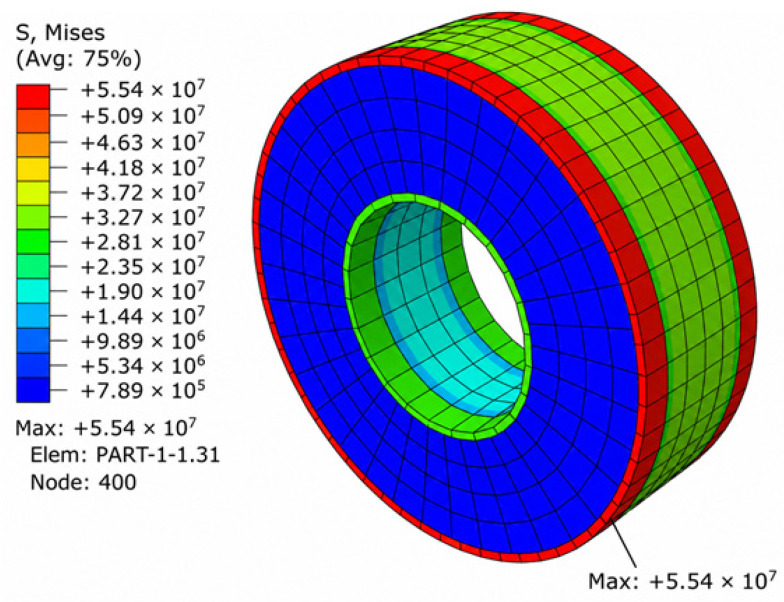
Distribution of von Mises equivalent stress at the time corresponding to the local cure-ramp peak circumferential stress.

To identify the region of stress concentration, the von Mises equivalent stress distribution at the time corresponding to the local cure-ramp peak circumferential stress is presented in Figure 10. The maximum von Mises equivalent stress is located near the outer circumferential edge of the outer steel ring, where the combined geometric constraint and thermal contraction mismatch produce a pronounced stress concentration. It should be noted that the von Mises stress is used here only to visualize the overall stress-concentration region. Because epoxy resin cracking and interfacial debonding depend on the stress components, fracture properties, and interfacial strength rather than solely on the von Mises equivalent stress, the contour cannot be directly interpreted as a prediction of cracking or debonding. In addition, material-specific failure data were not available in the present idealized example. The calculated stress is therefore used only for comparison among the different curing-dependent stiffness-factor assumptions.

Quantitative results are summarized in Table 3. The continuously evolving stiffness-factor model predicts a local cure-ramp compressive-stress peak of −37.62 MPa at 19.64 s and a final stress of −524.54 MPa. Because the steel layers were modeled as linearly elastic without a yielding criterion, the absolute final stress magnitude should be interpreted only as an idealized comparative result rather than as a realistic post-yield response of a specific steel grade. Replacing the evolving factor with constant values of 0.2, 0.4, and 0.8 changes the peak stress by −50.79%, −16.08%, and 35.91%, respectively, and changes the final stress by 56.35%, 34.47%, and 7.93%. The differences arise because a constant factor assigns the same load-carrying capability to material formed at different cure states, whereas the variable formulation continuously updates the contribution of the evolving network. Because the temperature and degree of cure are spatially uniform, the present example does not represent process-induced thermal or cure gradients. Coupling the constitutive model with heat conduction and cure kinetics to resolve non-uniform fields is required for process-level application.

The present sensitivity analyses consider the curing-dependent stiffness factor and cooling rate separately. Other parameters, including the cure rate, cure-shrinkage magnitude, relaxation spectrum, resin-layer thickness, and stiffness mismatch, may interact with these factors and should be investigated using experimentally calibrated material data. Consequently, the present results should not be interpreted as a complete process-optimization study.

### 4.2. Influence of Cooling Rate on the Predicted Circumferential Stress

To further investigate the influence of the prescribed process history on the response predicted by the proposed constitutive formulation, an additional cooling-rate sensitivity analysis was performed using the encapsulation model. The geometric configuration, mesh, interface constraints, material properties, degree-of-cure history, cure-shrinkage history, and curing-dependent stiffness functions were maintained identical to those of the baseline model. Only the duration of the cooling stage was varied. The baseline cooling duration of 60 s was increased to 120 s and reduced to 30 s, corresponding to relative cooling rates of 0.5q_0_, q_0_, and 2q_0_, respectively. All three cases were cooled through the same temperature interval.

**Figure 11:**
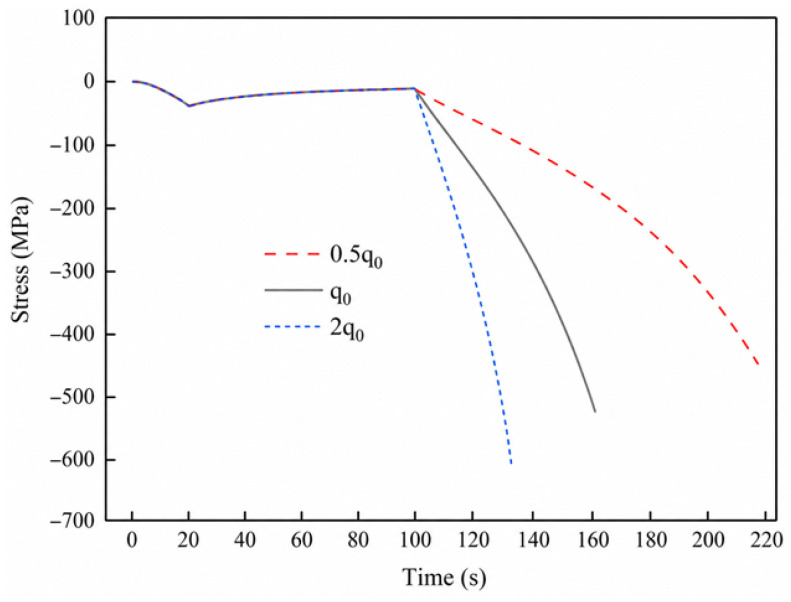
Effect of cooling rate on the predicted circumferential stress of the encapsulation model. The baseline cooling rate is denoted by q_0_, while 0.5q_0_ and 2q_0_ correspond to cooling durations of 120 and 30 s, respectively. The temperature interval, material parameters, cure history, shrinkage history, and interface constraints are identical in the three cases.

As shown in Figure 11, the three stress histories coincide before the onset of cooling at t = 100 s because their preceding temperature, cure, shrinkage, and mechanical histories are identical. Clear differences subsequently develop during the cooling stage. The final circumferential stresses obtained at 0.5q_0_, q_0_, and 2q_0_ are −439.932, −524.540, and −595.396 MPa, respectively. The corresponding quantitative results are summarized in Table 4.

Taking the baseline condition q_0_ as the reference, decreasing the cooling rate to 0.5q_0_ reduces the magnitude of the final compressive stress by 16.13%, whereas increasing the cooling rate to 2q_0_ increases it by 13.51%. This monotonic trend indicates that a lower cooling rate provides more time for viscoelastic relaxation, thereby reducing the accumulated compressive stress. In contrast, rapid cooling restricts stress relaxation while the constrained thermal contraction and temperature-dependent increase in stiffness occur over a shorter period, resulting in a larger final compressive-stress magnitude.

The additional analysis demonstrates that the predicted residual stress is sensitive not only to the curing-dependent stiffness assumption but also to the prescribed cooling history. Nevertheless, the results remain an idealized numerical sensitivity study because the temperature field is spatially uniform and the material parameters are not calibrated for a specific commercial epoxy system.

### 4.3. Comparison with Published Experimental Observations and Current Limitations

The preceding sensitivity analyses demonstrate that both the curing-dependent stiffness assumption and the prescribed cooling history significantly affect the calculated stress response. To assess whether these numerical trends are consistent with independently reported physical observations, relevant experimental studies in the literature are discussed below. De Vreugd et al. [45] measured the temperature-dependent warpage of a copper/epoxy-molding-compound bilayer and compared the measurements with several constitutive assumptions. Their cure-dependent viscoelastic model reproduced the measured warpage trend with an error band of approximately 15%, whereas the use of fully cured viscoelastic properties produced substantial overprediction when the full cure shrinkage was imposed and underprediction when cure shrinkage was omitted. These results demonstrate that cure shrinkage and the evolution of load-carrying properties cannot generally be reduced to a single constant initial-strain correction.

This published observation is qualitatively consistent with Figure 9 of the present study, in which different assumptions regarding the curing-dependent stiffness factor produce markedly different stress histories. However, the present calculations do not constitute a direct reproduction of the published warpage experiment. The complete material-specific Prony spectrum, cure-dependent shift-factor parameters, cure kinetics, and thermal history required for such a reproduction are not available in the published dataset. Therefore, the comparison is limited to physical consistency, and independent material-specific validation remains necessary before quantitative engineering application.

## 5. Conclusions

A curing-dependent viscoelastic constitutive framework was developed to describe the stress evolution of epoxy resin during curing. The progressive formation of the load-carrying polymer network was represented phenomenologically through cure-dependent equilibrium and Maxwell-branch stiffness functions. Based on this representation, a one-dimensional history-integral constitutive equation was derived and subsequently extended to non-isothermal conditions by incorporating temperature-dependent stiffness and reduced-time effects.

Explicit stress-increment equations were obtained for one-dimensional and three-dimensional isotropic materials and implemented in ABAQUS through a UMAT. Comparisons with direct evaluations of the constitutive equations showed small maximum absolute, final-time, and root-mean-square stress errors in both verification cases. These results demonstrate the numerical consistency of the derived stress-update algorithm and its UMAT implementation, rather than constituting independent physical validation of the material model.

The encapsulation example showed that the assumed curing-dependent stiffness factor has a pronounced influence on the calculated stress evolution. Relative to the continuously evolving stiffness-factor case, the constant-factor assumptions produced clear differences in the local cure-ramp peak circumferential stress, the time corresponding to the peak stress, and the final stress. The additional cooling-rate analysis further demonstrated the sensitivity of the predicted final stress to the prescribed process history. The final circumferential stresses obtained at 0.5q_0_, q_0_, and 2q_0_ were −439.932, −524.540, and −595.396 MPa, respectively. Relative to the baseline condition, slow cooling reduced the final compressive-stress magnitude by 16.13%, whereas fast cooling increased it by 13.51%. This trend indicates that slower cooling permits more extensive viscoelastic relaxation, while faster cooling restricts relaxation and increases the accumulated compressive stress. The von Mises stress distribution further identified the principal stress-concentration region under the prescribed constraints. The comparison with previously published warpage observations also indicates that the evolution of load-carrying properties during curing cannot generally be replaced by fully cured properties or a single constant correction. Nevertheless, the present encapsulation results constitute an illustrative sensitivity analysis and should not be interpreted as direct predictions of resin cracking or interfacial debonding.

The present study is limited by the use of idealized material parameters, spatially uniform temperature and degree-of-cure histories, and perfectly bonded interfaces. In addition, independent material-specific experimental validation has not yet been completed. The anisotropic equations presented in this work therefore constitute only a formal tensorial extension and still require direction-dependent parameter identification and numerical assessment. Future work should combine the proposed formulation with experimentally identified DSC and DMA data, non-uniform thermo-chemical fields, and independent measurements of cure-induced stress or deformation.

## Figures and Tables

**Table 1:** Idealized material parameters used exclusively for constitutive and UMAT verification.

Parameters	Value	Parameters	Value
$K_{0}^{\infty }$	2 GPa	$G_{0}^{\infty }$	100 MPa
$\bar{K}$	3 GPa	$\bar{G}$	1.2 GPa
n1	1	n2	1
$a_{K(T,\alpha )}$	(80 °C)/T	$a_{G(T,\alpha )}$	(80 °C)/T
$\tau_{K}$	8 s	$\tau_{G}$	10 s
$\chi_{K(\alpha )}$	α	$\chi_{G(\alpha )}$	α
$\psi_{K(\alpha )}$	α	$\psi_{G(\alpha )}$	α
$D_{K}(T)$	(80 °C)/T	$D_{G}(T)$	(80 °C)/T

**Table 2:** Quantitative errors between the direct constitutive calculations and UMAT results for the two verification cases.

Verification Case	Maximum Absolute Error (MPa)	Final-Stress Error (MPa)	RMSE (MPa)
Example 1	0.165	0.165	0.054
Example 2	2.299	0.262	0.597

**Table 3:** Quantitative comparison of the circumferential stress predicted using different curing-dependent stiffness-factor assumptions.

Stiffness-Factor Assumption	Local Cure-Ramp Peak Stress (MPa)	Time of Local Cure-Ramp Peak (s)	Final Stress (MPa)	Peak-Stress Error (%)	Final-Stress Error (%)
Variable, α	−37.62	19.64	−524.54	—	—
Constant 0.2	−18.51	19.64	−228.92	50.79	56.35
Constant 0.4	−31.57	19.64	−343.68	16.08	34.47
Constant 0.8	−51.13	19.64	−482.91	35.91	7.93

**Table 4:** Quantitative influence of cooling rate on the final circumferential stress of the encapsulation model.

Cooling Condition	Cooling Duration (s)	Relative Cooling Rate	Final Circumferential Stress (MPa)	Change in Final Compressive-Stress Magnitude Relative to q_0_
Slow cooling	120	0.5q_0_	−439.932	16.13% lower
Baseline cooling	60	q_0_	−524.540	Reference
Fast cooling	30	2q_0_	−595.396	13.51% higher

## Data Availability

The original contributions presented in this study are included in the article. Further inquiries can be directed to the corresponding authors.
